# Urinary tract infection in urolithiasis patients: risk factor analysis, pathogen distribution, and antimicrobial resistance characteristics

**DOI:** 10.3389/fpubh.2026.1888265

**Published:** 2026-08-05

**Authors:** Lingfei Yan, Zhuojie He, Tao Wang, Qing Li, Dawei Liu

**Affiliations:** Department of Urology, Fifth Affiliated Hospital of Southern Medical University, Guangzhou, Guangdong, China

**Keywords:** antimicrobial resistance, pathogen distribution, risk factors, urinary tract infection, urolithiasis

## Abstract

**Objective:**

This study investigated risk factors, pathogen distribution, and antimicrobial resistance characteristics of urinary tract infection (UTI) in patients with urolithiasis.

**Methods:**

A retrospective analysis was conducted among 934 patients with urolithiasis treated between April 2020 and April 2025. Patients were classified into a UTI group (*n* = 194) and a non-UTI group (*n* = 740). Clinical variables, stone-related characteristics, urinary parameters, renal function indicators, urine culture results, and antimicrobial susceptibility data were analyzed. Univariate analysis and multivariate logistic regression were used to identify independent risk factors for UTI.

**Results:**

Among 178 non-duplicate pathogenic isolates from the UTI group, Gram-negative bacteria predominated (68.54%), followed by Gram-positive bacteria (27.53%) and fungi (3.93%). *Escherichia coli* and *Pseudomonas aeruginosa* were the most common Gram-negative pathogens, while *Enterococcus faecalis* and *Enterococcus faecium* were the main Gram-positive pathogens. *Escherichia coli* showed high resistance to ampicillin, cefazolin, and ceftazidime, whereas no imipenem-resistant *Escherichia coli* or *Pseudomonas aeruginosa* isolates were detected. Multivariate logistic regression identified hydronephrosis, urinary tract obstruction, urine pH ≥ 7.0, stone diameter ≥3 mm, and serum creatinine ≥106 μmol/L as independent risk factors for UTI.

**Conclusion:**

Urolithiasis-associated UTI is mainly caused by Gram-negative bacteria with notable antimicrobial resistance. Early identification of high-risk patients, timely relief of obstruction, and antimicrobial therapy guided by susceptibility testing may help improve infection management.

## 1 Introduction

Urolithiasis is among the most common diseases encountered in urological practice, and its occurrence and progression are closely related to multiple factors, including metabolic abnormalities, changes in urinary composition, lifestyle, and environmental factors (1, 2). With the acceleration of population aging and changes in dietary structure and lifestyle, the prevalence of urinary calculi has shown a year-by-year increasing trend. Characterized by a high recurrence rate and prolonged disease course, urolithiasis have become an important condition affecting patients’ quality of life and the allocation of medical resources (3, 4). In the clinical management of urolithiasis, urinary tract infection is one of the most common and challenging complications. Long-term presence of calculi can cause persistent irritation to the urinary tract mucosa, impair normal urine drainage, and disrupt local urinary defense mechanisms, thereby creating favorable conditions for the colonization and proliferation of pathogenic microorganisms (5). Conversely, persistent infection may further alter the physicochemical environment of urine, promote stone formation, or exacerbate existing disease, increasing the difficulty of treatment (6). Clinical practice has shown that once urolithiasis are complicated by UTI, patients not only experience aggravated symptoms, but also significantly prolonged hospital stays and increased medical costs; in severe cases, infection may spread and even become life-threatening (7, 8).

The occurrence of UTI is closely associated with a variety of pathogenic microorganisms, and its etiological composition is influenced by multiple factors such as regional differences, patients’ baseline conditions, and medical environments.

Previous studies (9, 10) have demonstrated that Gram-negative bacteria are detected at a relatively high rate in urinary system-related infections; however, in recent years, the proportions of Gram-positive bacterial and fungal infections have also shown an increasing trend. Meanwhile, the long-term and inappropriate use of antimicrobial agents has led to increasingly prominent antimicrobial resistance, with continuously changing resistance patterns, posing uncertainty for empirical anti-infective therapy in clinical practice (11, 12). In the absence of regional etiological data, blind selection of antimicrobial agents may result in poor therapeutic efficacy or even delayed treatment.

In addition to etiological factors, patients’ own conditions and disease-related characteristics are also considered to play a role in the development of UTI. However, findings regarding relevant influencing factors vary across studies, with some conclusions remaining controversial. Moreover, many studies have been limited by small sample sizes and have failed to comprehensively integrate pathogen distribution with antimicrobial resistance characteristics. Therefore, it is necessary to further explore the factors associated with UTI in patients with urinary calculi within a real-world clinical setting. Therefore, evaluating UTI-related clinical factors, pathogen composition, and antimicrobial resistance patterns in patients with urolithiasis may provide useful evidence for infection prevention, antimicrobial selection, and clinical management.

## 2 Data and methods

### 2.1 Study design and ethics

This study adopted a retrospective observational design based on real-world clinical data from a single center. Study data were obtained from the hospital’s electronic medical record system, laboratory information system, and picture archiving and communication system (PACS). Through systematic retrieval, medical records of patients diagnosed with urinary system calculi who were treated at our hospital between April 2020 and April 2025 were identified, and the clinical data of eligible patients were collated and analyzed. To reduce information bias, a standardized data collection form was developed prior to data extraction. Two investigators independently performed data extraction and entry, followed by cross-checking after completion. Any discrepancies were resolved by re-examining the original medical records. The study was conducted in accordance with the Declaration of Helsinki and was approved by the Medical Ethics Committee of The Fifth Affiliated Hospital, Southern Medical University (Approval No.: 25SZYJYKLC-02). Because this was a retrospective study using anonymized clinical data and routine clinical care was not affected, the requirement for written informed consent was waived by the ethics committee. All included clinical data were anonymized before analysis to protect patient privacy, and no potentially identifiable personal information or images were included.

### 2.2 Screening process of study participants

**Figure 1:**
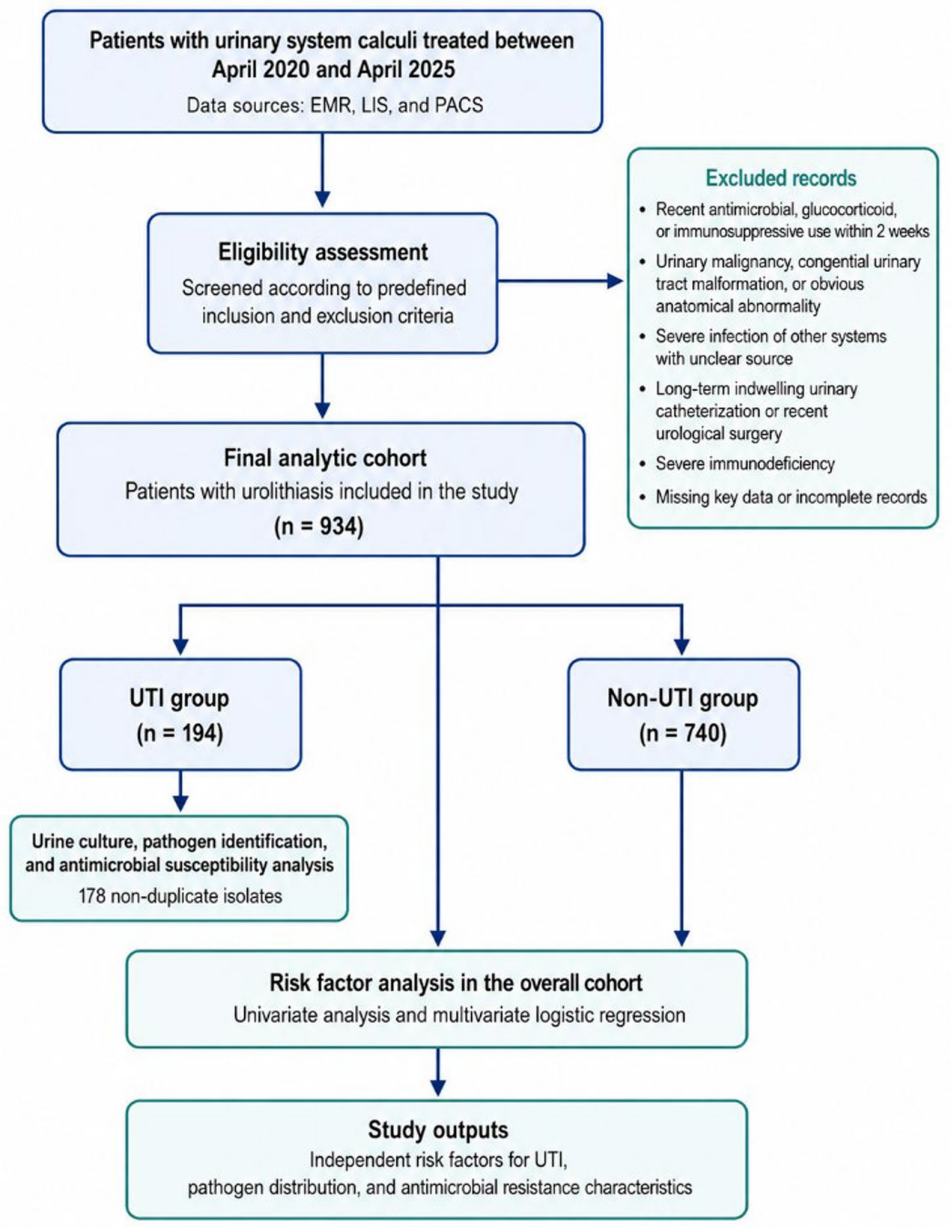
Flow diagram of patient screening, grouping, and study analysis Patients with imaging-confirmed urolithiasis treated between April 2020 and April 2025 were screened according to predefined inclusion and exclusion criteria. The final analytic cohort included 934 patients, including 194 patients with UTI and 740 patients without UTI. Pathogen distribution and antimicrobial resistance analyses were performed in the infection group, while risk factor analysis was conducted in the overall cohort.

The screening of study participants was conducted in three steps: (1) initial identification of all patients with urolithiasis based on diagnostic codes and imaging findings; (2) further screening of preliminarily identified cases according to the inclusion and exclusion criteria; and (3) exclusion of cases with missing key data or incomplete information. Cases with missing core variables required for grouping, urine culture interpretation, imaging assessment, or major risk factor analysis were excluded from the final analytic dataset. After this screening process, a total of 934 patients with urolithiasis were ultimately included in the study. According to whether urinary tract infection occurred during diagnosis and treatment, patients were divided into an infection group (n = 194) and a non-infection group (n = 740). Grouping was strictly performed based on predefined diagnostic criteria to avoid subjective bias. A brief overview of the screening process is shown in Figure 1.

### 2.3 Inclusion and exclusion criteria

Inclusion criteria: (1) age ≥18 years, with no restriction on sex; (2) diagnosis of urolithiasis confirmed by imaging examinations [urinary ultrasound, computed tomography (CT), or X-ray]; (3) first-time diagnosis, with calculi involving either the upper or lower urinary tract; (4) no use of glucocorticoids, immunosuppressive agents, or antimicrobial drugs within 2 weeks prior to urine testing, to minimize the influence of recent antimicrobial exposure on urine culture and susceptibility results; (5) complete clinical records, allowing access to basic demographic information, imaging data, and laboratory test results.

Exclusion criteria: (1) presence of urinary system malignancies, congenital urinary tract malformations, or obvious anatomical abnormalities; (2) concomitant severe infections of other systems during the study period with unclear infection sources; (3) long-term indwelling urinary catheterization or recent (within 1 month prior to enrollment) urological surgery; (4) severe immunodeficiency conditions (e.g., long-term use of immunosuppressants, post-organ transplantation); (5) missing key study variables or incomplete records.

### 2.4 Diagnostic criteria and grouping of urinary tract infection

The diagnosis of urinary tract infection was comprehensively determined based on clinical manifestations, laboratory findings, and microbiological results, in accordance with relevant clinical guidelines (13). Diagnostic criteria included: (1) presence of one or more clinical symptoms such as urinary frequency, urgency, dysuria, fever, or flank pain; (2) leukocyte count >10 per high-power field (HPF) in midstream urine sediment, and quantitative midstream urine culture showing Gram-negative bacilli >1 × 10^5^ colony-forming units per milliliter (CFU/mL) or Gram-positive cocci >1 × 10^4^ CFU/mL. Patients meeting the above criteria were diagnosed with urinary tract infection and assigned to the infection group, while those not meeting the criteria were assigned to the non-infection group.

### 2.5 Observational indicators and variable definitions

Observational indicators in this study were established based on patients’ general characteristics, disease-related features, and laboratory test results. (1) General demographic indicators: sex, age, body mass index (BMI), etc.; (2) Stone-related indicators: stone location, maximum stone diameter (based on the maximum diameter measured in imaging reports), and presence of multiple calculi; Stone composition was not included as an analytical variable because standardized stone component testing was not routinely available for all patients during the retrospective study period. (3) Imaging indicators: presence of hydronephrosis and urinary tract obstruction, as determined by imaging findings; (4) Laboratory indicators: urine pH value, routine urinalysis, and relevant parameters from routine blood tests; (5) Microbiological indicators (infection group): urine culture results, pathogen species, and antimicrobial susceptibility results.

For statistical convenience, some continuous variables were categorized according to clinical practice and the available retrospective data. For stone diameter, the cutoff of ≥3 mm was used as a study-specific imaging-based categorization threshold rather than a universally accepted clinical intervention threshold.

### 2.6 Pathogen culture, identification, and antimicrobial resistance analysis

Specimen collection methods for patients with urolithiasis complicated by urinary tract infection included: (1) prior to treatment, after cleansing the external genitalia, patients voided spontaneously and midstream urine samples were collected; (2) after identifying the stone location, disinfection and draping were performed, a cystoscope was inserted to obtain bladder urine, and approximately 15 mL of midstream urine was collected in sterile tubes and immediately sent for examination.

Detection procedures were as follows: midstream urine samples were centrifuged at 3,000–4,000 r/min for 30 min, the supernatant was discarded, and the sediment was inoculated onto MacConkey agar plates and blood agar plates (Shanghai Guangrui Biotechnology Co., Ltd.), followed by incubation at 35 °C for 16–24 h. An automated colony counter (Interscience Scan 1,200, Shanghai Dibos Biotechnology Co., Ltd.) and an automated microbial identification system (Thermo Scientific, Thermo Fisher Scientific Inc., United States) were used to determine pathogen species and quantities.

Antimicrobial susceptibility testing was performed using the minimum inhibitory concentration method. Resistance rate was calculated as the number of resistant strains divided by the total number of strains × 100%. Antimicrobial susceptibility testing was performed using the minimum inhibitory concentration (MIC) method. Resistance rates were calculated as the number of resistant strains divided by the total number of tested strains × 100%. Antimicrobial susceptibility results were interpreted according to the breakpoints recommended by the Clinical and Laboratory Standards Institute (CLSI M100, 28th edition, 2018) (14). Because this was a retrospective study covering several calendar years, the original susceptibility interpretations recorded in the laboratory information system were retained to ensure consistency across the entire study period. Updated CLSI breakpoints were not applied retrospectively because complete isolate-level MIC values and antimicrobial panel details required for uniform re-interpretation were not available for all historical isolates. Quality control was performed using American Type Culture Collection (ATCC) reference strains, including *Escherichia coli* ATCC 25922, *Klebsiella pneumoniae* ATCC 700603, *Pseudomonas aeruginosa* ATCC 27853, *Staphylococcus aureus* ATCC 25923, and *Enterococcus faecalis* ATCC 29212. All quality control results were within the acceptable ranges specified by CLSI, and only susceptibility results from tests that passed quality control were included in the final analysis. In addition to single-drug resistance rates, multidrug resistance (MDR), extended-spectrum *β*-lactamase (ESBL)-producing Enterobacterales, and carbapenem-resistant organisms (CROs) were recorded when available from microbiology reports. MDR was defined as acquired non-susceptibility to at least one agent in three or more antimicrobial categories. ESBL-producing Enterobacterales and CROs were identified according to laboratory susceptibility reports and CLSI-based interpretation. To further evaluate temporal changes in antimicrobial resistance, annual resistance rates were descriptively analyzed for the two most frequently isolated Gram-negative pathogens, *Escherichia coli* and *Pseudomonas aeruginosa*, against representative antimicrobial agents. Because the number of isolates in each year was limited, especially in 2025, no formal time-series regression model was applied.

### 2.7 Statistical analysis methods

Statistical analyses were performed using Statistical Package for the Social Sciences (SPSS) version 26.0. Categorical data were expressed as number of cases (%), and comparisons between groups were conducted using the *χ*^2^ test or Fisher’s exact test (when theoretical frequencies were <5). The occurrence of urinary tract infection was used as the dependent variable, and potential related clinical factors were analyzed using univariate analysis. Variables with statistically significant differences were subsequently included in a multivariate Logistic regression model to identify factors associated with urinary tract infection in patients with urolithiasis. Odds ratios (ORs) and 95% confidence intervals (95% CIs) were calculated. A *p* value <0.05 was considered statistically significant. Missing data were assessed before statistical analysis. After excluding cases with incomplete key information during screening, the final analytic cohort contained complete data for the main variables included in the univariate and multivariate analyses, including UTI status, urine culture results, stone characteristics, hydronephrosis, urinary tract obstruction, urine pH, serum urea nitrogen, and serum creatinine. Therefore, the missing rates of these key variables were 0%, complete-case analysis was performed, and no statistical imputation method was applied. Because this was a retrospective observational study, the sample size was determined by the number of consecutive eligible patients during the predefined study period rather than by a prospective power calculation. The number of UTI events was considered sufficient for the planned multivariate logistic regression analysis according to the events-per-variable principle.

## 3 Results

### 3.1 Baseline characteristics of patients with Urolithiasis

A total of 934 patients with urolithiasis were enrolled in this study (baseline characteristics are shown in Table 1). Of these, 428 patients were male (45.82%) and 506 were female (54. 18%), with a slightly higher proportion of females than males.

**Table 1:** Baseline characteristics of patients with urolithiasis.

Baseline characteristics	Number of cases (*n* = 934)	Proportion (%)
Sex		
Male	428	45.82
Female	506	54.18
Age (years)		
<60	558	59.74
≥60	376	40.26
BMI (kg/m^2^)		
<24	531	56.85
≥24	403	43.15
Hydronephrosis		
Yes	381	40.79
No	553	59.21
Urinary tract obstruction		
Yes	352	37.69
No	582	62.31
Urine pH value		
<7.0	633	67.77
≥7.0	301	32.23
Number of stones		
<2	575	61.56
≥2	359	38.44
Stone diameter (mm)		
<3	503	53.86
≥3	431	46.14
Stone location		
Upper urinary tract calculi	642	68.74
Lower urinary tract calculi Serum urea nitrogen (mmol/L)	292	31.26
<7.5	666	71.31
≥7.5	268	28.69
Serum creatinine (μmol/L)		
<106	615	65.84
≥106	319	34.16

BMI, body mass index.

Regarding age distribution, 558 patients (59.74%) were younger than 60 years, while 376 patients (40.26%) were aged 60 years or older, indicating that urolithiasis was common in both middle-aged and older adult populations. In terms of body weight status, 403 patients (43. 15%) had a body mass index (BMI) ≥ 24 kg/m^2^, suggesting that overweight was relatively prevalent in this population. Imaging and clinical findings showed that 381 patients (40.79%) had hydronephrosis and 352 patients (37.69%) had urinary tract obstruction, indicating that a substantial proportion of patients experienced impaired urinary drainage. Urinalysis revealed that 301 patients (32.23%) had a urinary pH ≥ 7.0. With respect to stone characteristics, 431 patients (46. 14%) had stones with a diameter ≥ 3 mm, and 359 patients (38.44%) had two or more stones. Stones were predominantly located in the upper urinary tract, accounting for 642 cases (68.74%). Renal function indicators showed that 268 patients (28.69%) had serum urea nitrogen levels ≥ 7.5 mmol/L, and 319 patients (34. 16%) had serum creatinine levels ≥ 106 μmol/L, indicating that a proportion of patients had varying degrees of renal function impairment.

### 3.2 Distribution of pathogens in patients with urolithiasis complicated by urinary tract infection

**Figure 2:**
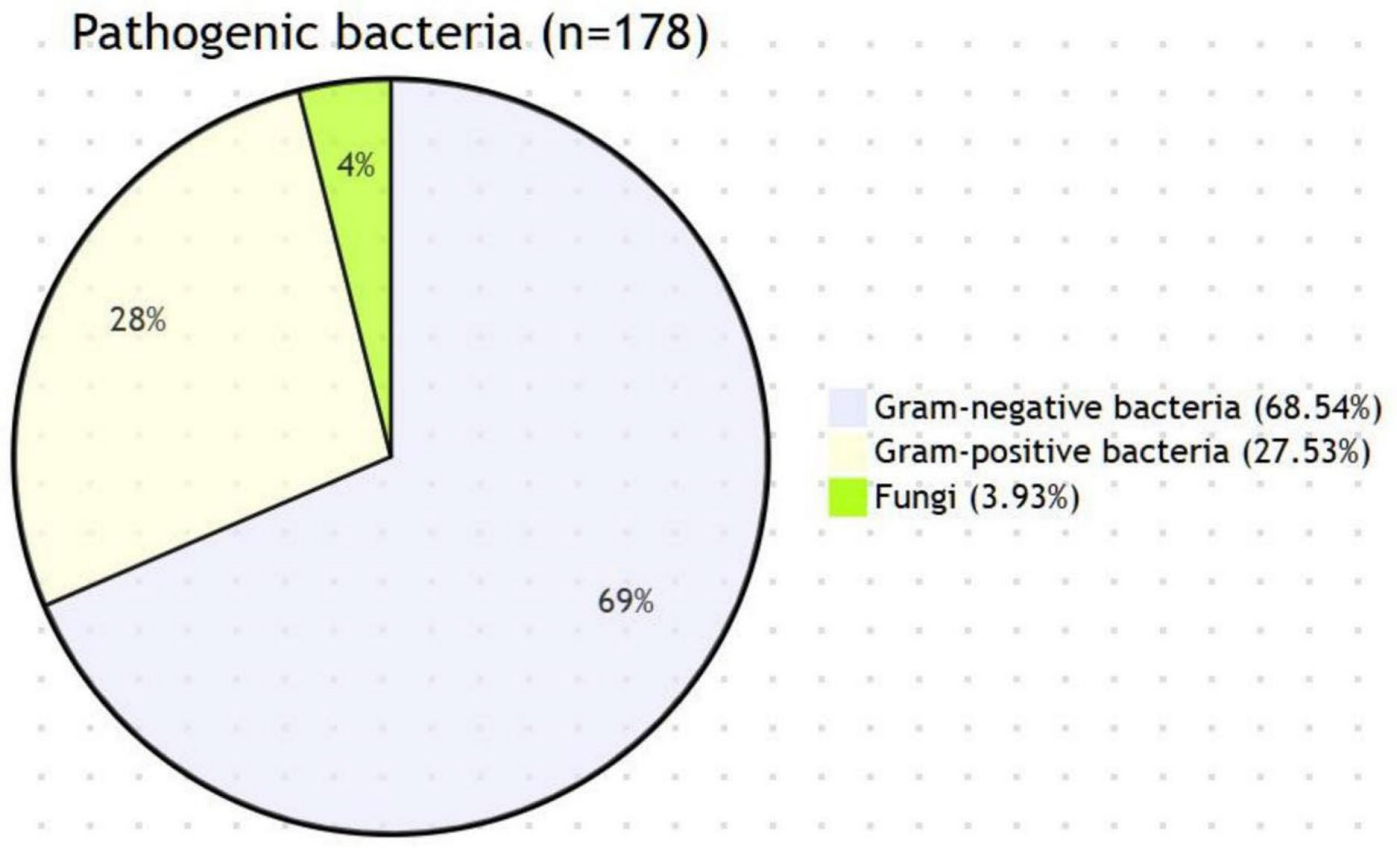
Pathogen distribution in patients with urolithiasis complicated by UTI among 178 non-duplicate pathogenic isolates, Gram-negative bacteria accounted for 68.54%, Gram-positive bacteria accounted for 27.53%, and fungi accounted for 3.93%.

Among patients in the infection group (*n* = 194), a total of 178 pathogenic isolates were identified after excluding duplicate strains. Overall pathogen composition showed that Gram-negative bacteria were predominant, with 122 isolates (68.54%), representing the major causative pathogens of urinary tract infection associated with urolithiasis. Gram-positive bacteria accounted for 49 isolates (27.53%), while fungi were relatively uncommon, with only 7 isolates (3.93%), indicating that fungal infections were rare in this study population (Table 2; Figure 2). Among Gram-negative bacteria, *Escherichia coli* was the most frequently isolated pathogen (41 isolates, 23.03%), followed by *Pseudomonas aeruginosa* (30 isolates, 16.85%).

**Table 2:** Distribution of pathogens in patients with urinary system calculi complicated by urinary tract infection.

Pathogen	Number of strains (*n* = 178)	Proportion (%)
Gram-negative bacteria	122	68.54
*Escherichia coli*	41	23.03
*Pseudomonas aeruginosa*	30	16.85
*Klebsiella pneumoniae*	19	10.67
*Acinetobacter baumannii*	18	10.11
*Enterobacter cloacae*	7	3.93
Others	7	3.93
Gram-positive bacteria	49	27.53
*Enterococcus faecalis*	25	14.04
*Enterococcus faecium*	9	5.06
*Staphylococcus aureus*	10	5.62
Others	5	2.81
Fungi	7	3.93
*Candida albicans*	4	2.25
*Candida tropicalis*	3	1.69
Total	178	100.00

Together, these two pathogens accounted for 39.88% of all isolates, highlighting their dominant role in urolithiasis-associated urinary tract infections. In addition, *Klebsiella pneumoniae* (19 isolates, 10.67%) and *Acinetobacter baumannii* (18 isolates, 10. 11%) were also commonly identified, suggesting that some patients may have had complicated infections or hospital-acquired infections. Although *Enterobacter cloacae* and other Gram-negative bacteria were less prevalent, they reflected the diversity of the pathogen spectrum. Among Gram-positive bacteria, Enterococcus species predominated, with *Enterococcus faecalis* and *Enterococcus faecium* together accounting for 69.39% of Gram-positive isolates. *Staphylococcus aureus* accounted for a relatively small proportion. Fungal infections were mainly caused by *Candida albicans*, followed by *Candida tropicalis*.

### 3.3 Antimicrobial resistance analysis of major gram-negative bacteria in patients with urolithiasis complicated by urinary tract infection

**Figure 3:**
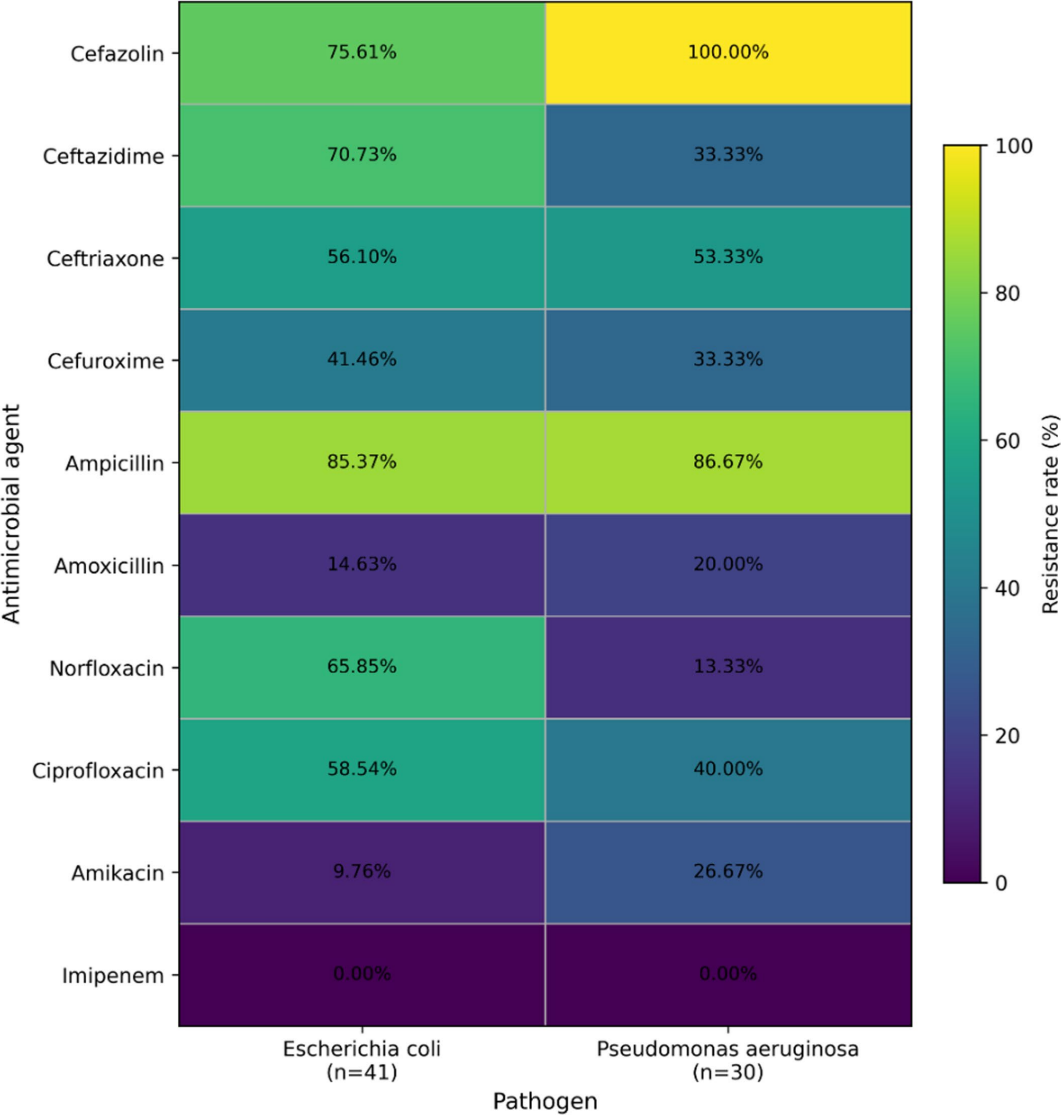
Heatmap of antimicrobial resistance profiles of major gram-negative pathogens in patients with urolithiasis complicated by UTI the heatmap shows resistance rates of *Escherichia coli* and *Pseudomonas aeruginosa* to commonly tested antimicrobial agents. Darker cells indicate higher resistance rates.

Antimicrobial resistance analysis demonstrated marked differences in resistance patterns among major Gram-negative pathogens; however, an overall trend of high resistance to commonly used first-line antibiotics was observed (Table 3; Figure 3). Among *Escherichia coli* isolates (*n* = 41), the highest resistance rate was observed for ampicillin (85.37%, 35/41). Resistance rates to cefazolin and ceftazidime were 75.61% (31/41) and 70.73% (29/41), respectively, indicating substantial resistance to *β*-lactam antibiotics. Moderate to high resistance rates (approximately 55–65%) were also observed for third-generation cephalosporins and fluoroquinolones, suggesting potential limitations of empirical antimicrobial therapy in this population.

**Table 3:** Antimicrobial resistance analysis of major gram-negative bacteria in patients with urolithiasis complicated by urinary tract infection.

Antibiotic	Gram-negative bacteria	
	*Escherichia coli* (*n* = 41)	*Pseudomonas aeruginosa* (*n* = 30)
Cefazolin	31 (75.61%)	30 (100.00%)
Ceftazidime	29 (70.73%)	10 (33.33%)
Ceftriaxone	23 (56. 10%)	16 (53.33%)
Cefuroxime	17 (41.46%)	10 (33.33%)
Ampicillin	35 (85.37%)	26 (86.67%)
Amoxicillin	6 (14.63%)	6 (20.00%)
Norfloxacin	27 (65.85%)	4 (13.33%)
Ciprofloxacin	24 (58.54%)	12 (40.00%)
Amikacin	4 (9.76%)	8 (26.67%)
Imipenem	0 (0.00%)	0 (0.00%)

UTI, urinary tract infection.

In contrast, *Escherichia coli* showed a low resistance rate to amikacin (9.76%, 4/41), and no imipenem-resistant isolates were detected. *Pseudomonas aeruginosa* (*n* = 30) exhibited more pronounced multidrug-resistant characteristics. All isolates were resistant to cefazolin (100.00%, 30/30), and high resistance rates were also observed for multiple cephalosporins and penicillin antibiotics. Although resistance rates to fluoroquinolones were lower than those observed for *E. coli*, a considerable proportion of resistant isolates was still present. Notably, *P. aeruginosa* remained highly susceptible to imipenem, highlighting the important clinical value of carbapenems in the treatment of complicated or severe urinary tract infections. Because isolate-level MDR and ESBL confirmation data were not completely available in the retrospective microbiology records, formal MDR and ESBL prevalence could not be reliably calculated. However, the high resistance rates of *Escherichia coli* and *Klebsiella pneumoniae* to cephalosporins suggested the need for continued surveillance of ESBL-producing Enterobacterales. No imipenem-resistant *Escherichia coli* or *Pseudomonas aeruginosa* isolates were detected, suggesting that carbapenem resistance was uncommon among the major pathogens analyzed.

### 3.4 Antimicrobial resistance analysis of major gram-positive bacteria in patients with urolithiasis complicated by urinary tract infection

**Figure 4:**
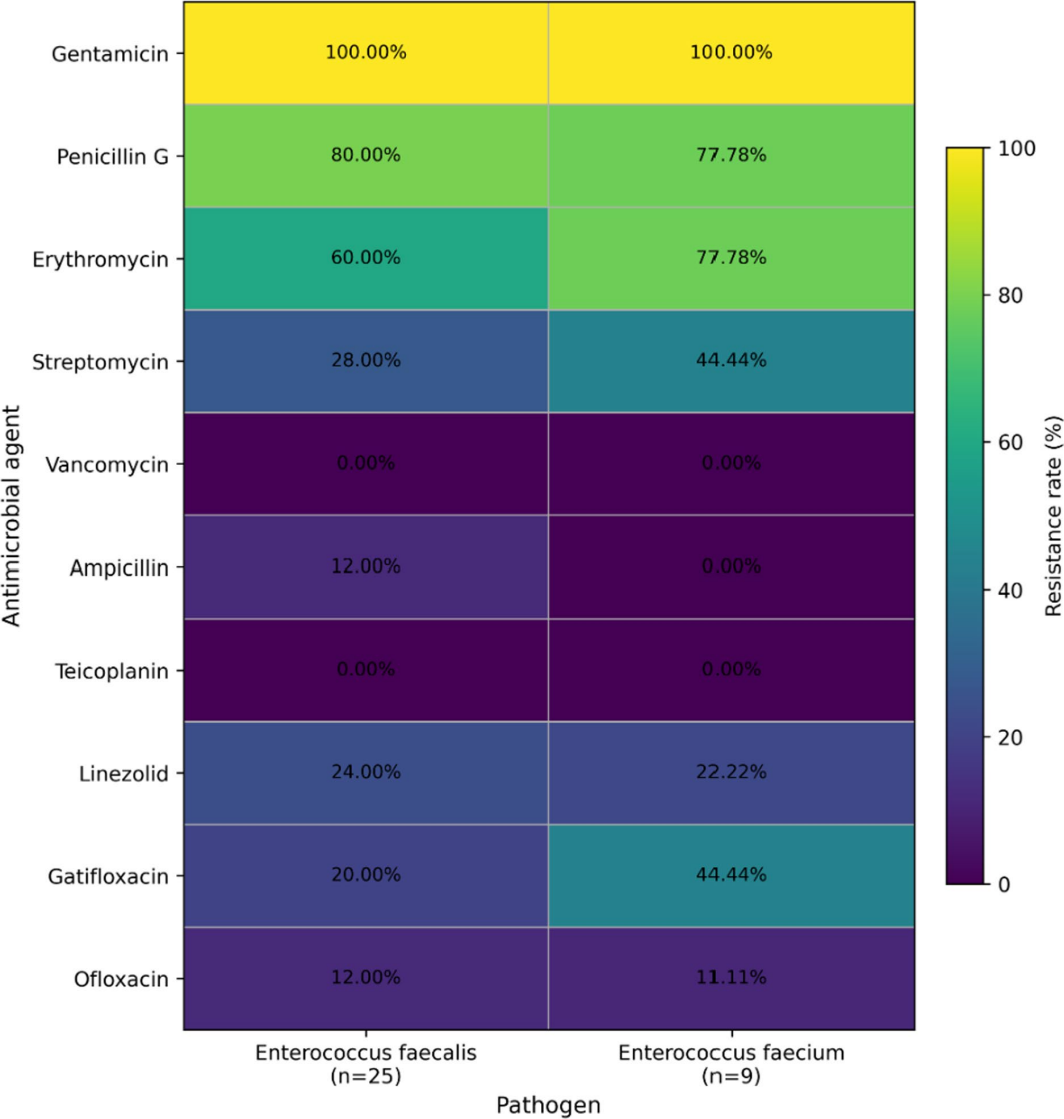
Heatmap of antimicrobial resistance profiles of major Gram-positive pathogens in patients with urolithiasis complicated by UTI the heatmap shows resistance rates of *Enterococcus faecalis* and *Enterococcus faecium* to commonly tested antimicrobial agents. Darker cells indicate higher resistance rates.

Analysis of antimicrobial resistance among Gram-positive bacteria showed that Enterococcus species remained the predominant Gram-positive pathogens in patients with urolithiasis complicated by urinary tract infection. Among them, *Enterococcus faecalis* (*n* = 25) and *Enterococcus faecium* (*n* = 9) were the dominant species (Table 4; Figure 4). Antimicrobial susceptibility profiles demonstrated that both *Enterococcus faecalis* and *E. faecium* exhibited high resistance to multiple commonly used antibiotics.

**Table 4:** Antimicrobial resistance analysis of major gram-positive bacteria in patients with urolithiasis complicated by urinary tract infection.

Antibiotic	Gram-positive bacteria	
	*Enterococcus faecalis* (*n* = 25)	*Enterococcus faecium* (*n* = 9)
Gentamicin	25 (100.00%)	9 (100.00%)
Penicillin G	20 (80.00%)	7 (77.78%)
Erythromycin	15 (60.00%)	7 (77.78%)
Streptomycin	7 (28.00%)	4 (44.44%)
Vancomycin	0 (0.00%)	0 (0.00%)
Ampicillin	3 (12.00%)	0 (0.00%)
Teicoplanin	0 (0.00%)	0 (0.00%)
Linezolid	6 (24.00%)	2 (22.22%)
Gatifloxacin	5 (20.00%)	4 (44.44%)
Ofloxacin	3 (12.00%)	1 (11. 11%)

Specifically, resistance to gentamicin reached 100% in both species, indicating that high-level aminoglycoside resistance was prevalent in these infections and that the clinical efficacy of aminoglycosides was markedly limited. In addition, high resistance rates to penicillin G and erythromycin were observed in both species, highlighting significant resistance to traditional *β*-lactam and macrolide antibiotics.

Some isolates also showed varying degrees of resistance to fluoroquinolones, suggesting a potential risk of treatment failure during empirical therapy. In contrast, no resistance to vancomycin or teicoplanin was detected in either *Enterococcus faecalis* or *E. faecium*, and high susceptibility to linezolid was maintained. These findings indicate that glycopeptide antibiotics and oxazolidinones remain important and reliable therapeutic options for the treatment of enterococcal urinary tract infections.

### 3.5 Temporal trends in antimicrobial resistance from 2020 to 2025

**Figure 5:**
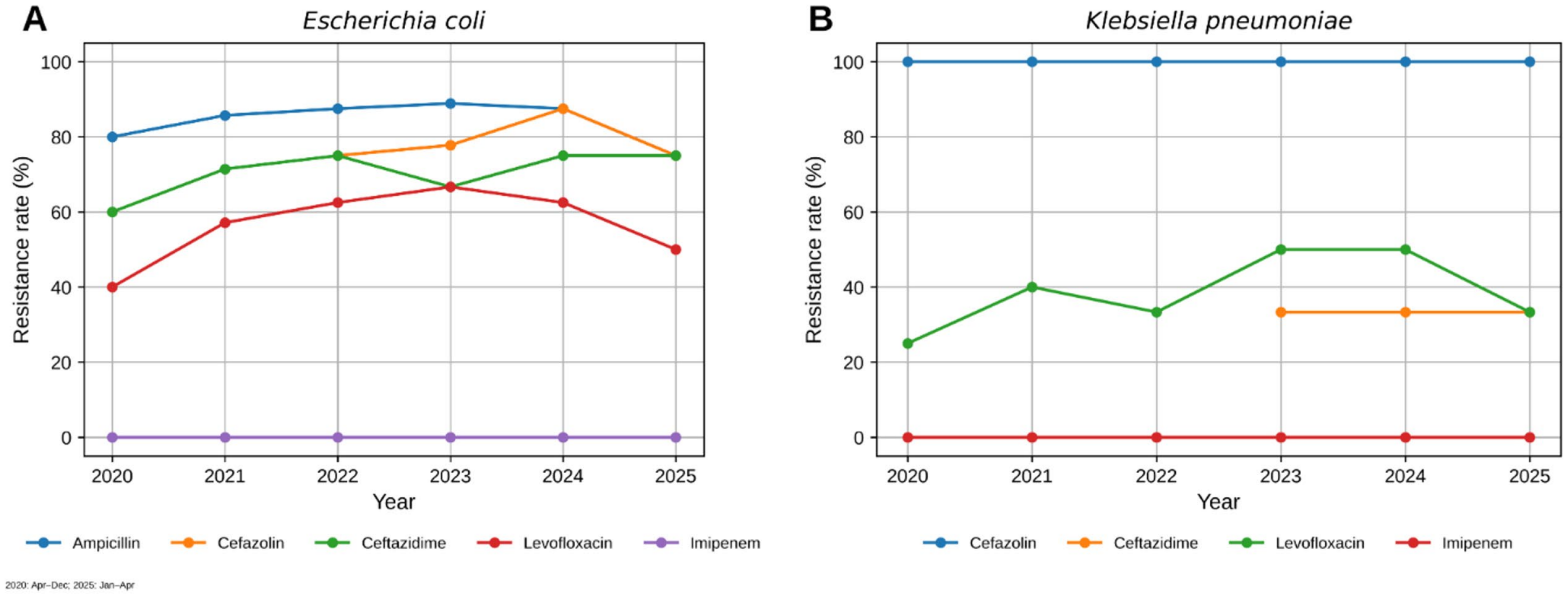
Annual resistance trends of major gram-negative pathogens from 2020 to 2025 temporal resistance trend analysis showed that *Escherichia coli* maintained persistently high resistance to ampicillin, cefazolin, and ceftazidime from 2020 to 2025, whereas resistance to levofloxacin fluctuated over time. No imipenem-resistant *Escherichia coli* isolates were detected. In Klebsiella pneumoniae, cefazolin resistance remained consistently 100% throughout the study period, while resistance to ceftazidime and levofloxacin fluctuated moderately. No imipenem-resistant Klebsiella pneumoniae isolates were identified.

To further assess changes in antimicrobial resistance during the study period, annual resistance rates of the two most frequently isolated Gram-negative pathogens, *Escherichia coli* and *Pseudomonas aeruginosa*, were analyzed descriptively (Figure 5). For *Escherichia coli*, resistance to ampicillin remained consistently high throughout the study period, ranging from 75.00 to 88.89%. Resistance to cefazolin and ceftazidime also remained at relatively high levels, with a slight increase observed in cefazolin resistance from 60.00% in 2020 to 87.50% in 2024. Fluoroquinolone resistance, represented by ciprofloxacin, fluctuated between 40.00 and 66.67%. No imipenem-resistant *Escherichia coli* isolates were detected during the study period. For *Pseudomonas aeruginosa*, resistance to cefazolin remained 100.00% across all years. Resistance rates to ceftazidime and ciprofloxacin fluctuated over time but showed no marked continuous upward trend. Similar to *Escherichia coli*, no imipenem-resistant *Pseudomonas aeruginosa* isolates were identified. Overall, these findings suggest persistently high resistance to several commonly used *β*-lactam antibiotics, whereas carbapenem susceptibility was largely preserved among the major Gram-negative pathogens in this cohort.

### 3.6 Univariate analysis of clinical data between patients with and without urinary tract infection

Univariate analysis revealed significant differences in multiple clinical and laboratory parameters between patients with urolithiasis complicated by urinary tract infection and those without infection (Table 5). With respect to general characteristics, sex distribution did not differ significantly between the infection and non-infection groups (*p* = 0.140). Although the proportion of female patients was slightly higher in the infection group than in the non-infection group (57.73% vs. 53.24%), this difference was not statistically significant. No significant differences were observed between the two groups in terms of age distribution or body mass index (BMI) (*p* > 0.05). Regarding imaging and clinical findings, the incidence of hydronephrosis was significantly higher in the infection group than in the non-infection group (57.22% vs. 36.49%, *p* < 0.001). Similarly, urinary tract obstruction was more frequently observed in the infection group (53.61% vs. 33.24%, *p* < 0.001). Urinalysis showed that the proportion of patients with urinary pH ≥ 7.0 was significantly higher in the infection group compared with the non-infection group (51.55% vs. 27. 16%, *p* < 0.001). In terms of stone characteristics, a stone diameter ≥ 3 mm was more common in the infection group than in the non-infection group (56.70% vs. 43.38%, *p* < 0.001). No significant difference was found in stone location between the infection and non-infection groups. Upper urinary tract stones accounted for 68.56% of cases in the infection group and 68.78% in the non-infection group, while lower urinary tract stones accounted for 31.44 and 31.22%, respectively (*p* = 0.913). This finding suggested that, in the present cohort, UTI risk did not differ significantly between patients with upper and lower urinary tract stones. Regarding renal function indicators, the proportion of patients with serum urea nitrogen ≥ 7.5 mmol/L was significantly higher in the infection group than in the non-infection group (40.21% vs. 25.68%, *p* < 0.001).

**Table 5:** Univariate analysis of clinical data between patients with urinary system calculi with and without urinary tract infection.

Variable	Infection group (*n* = 194)	Non-infection group (*n* = 740)	2*x*	*p*
Sex			2.216	0.140
Male	82 (42.27%)	346 (46.76%)	-	-
Female	112 (57.73%)	394 (53.24%)	-	-
Age (years)			0.842	0.359
<60	114 (58.76%)	444 (60.00%)	-	-
≥60	80 (41.24%)	296 (40.00%)	-	-
BMI (kg/m^2^)			0.112	0.738
<24	109 (56. 19%)	422 (57.03%)	-	-
≥24	85 (43.81%)	318 (42.97%)	-	-
Hydronephrosis			28.906	< 0.001
Yes	111 (57.22%)	270 (36.49%)	-	-
No	83 (42.78%)	470 (63.51%)	-	-
Urinary tract obstruction			26.184	< 0.001
Yes	104 (53.61%)	246 (33.24%)	-	-
No	90 (46.39%)	494 (66.76%)	-	-
Urine pH			33.427	< 0.001
<7.0	94 (48.45%)	539 (72.84%)	-	-
≥7.0	100 (51.55%)	201 (27. 16%)	-	-
Number of stones			0.496	0.481
<2	118 (60.82%)	457 (61.76%)	-	-
≥2	76 (39. 18%)	283 (38.24%)	-	-
Stone diameter (mm)			10.942	< 0.001
<3	84 (43.30%)	419 (56.62%)	-	-
≥3	110 (56.70%)	321 (43.38%)	-	-
Stone location			0.012	0.913
Upper urinary tract calculi	133 (68.56%)	509 (68.78%)	-	-
Lower urinary tract calculi	61 (31.44%)	231 (31.22%)	-	-
Serum urea nitrogen (mmol/L)			15.216	< 0.001
<7.5	116 (59.79%)	550 (74.32%)	-	-
≥7.5	78 (40.21%)	190 (25.68%)	-	-
Serum creatinine (μmol/L)			18.904	< 0.001
<106	102 (52.58%)	514 (69.46%)	-	-
≥106	92 (47.42%)	226 (30.54%)	-	-

Likewise, elevated serum creatinine levels (≥ 106 μmol/L) were more frequently observed in the infection group than in the non-infection group (47.42% vs. 30.54%, *p* < 0.001).

### 3.7 Multivariate logistic regression analysis of risk factors for urinary tract infection in patients with urolithiasis

The occurrence of urinary tract infection in patients with urolithiasis was used as the dependent variable (coded as: no = 0, yes = 1). Variables with statistically significant differences in the univariate analysis (Table 5) were included as independent variables and assigned values accordingly (Table 6) for multivariate logistic regression analysis (Table 7). The results demonstrated that hydronephrosis, urinary tract obstruction, urine pH ≥ 7.0, calculus diameter ≥3 mm, and serum creatinine ≥106 μmol/L were all confirmed as independent risk factors for urinary tract infection in patients with urinary system calculi. Specifically, patients with hydronephrosis had a 2.36-fold higher risk of developing urinary tract infection compared with those without hydronephrosis (OR = 2.36, 95% CI: 1.34–4.17, *p* = 0.003), while the presence of urinary tract obstruction increased the infection risk to 2.03 times (OR = 2.03, 95% CI: 1.20–3.43, *p* = 0.009). Regarding urinary environmental factors, patients with urine pH ≥ 7.0 had a 2.56-fold higher risk of infection than those with urine pH < 7.0 (OR = 2.56, 95% CI: 1.48–4.45, *p* = 0.001). In addition, patients with a stone diameter ≥3 mm had an increased infection risk of 1.97 times (OR = 1.97, 95% CI: 1.17–3.33, *p* = 0.010). However, this threshold should be interpreted cautiously because it was used as a study-specific categorization based on retrospective imaging records, rather than as a universal clinical cutoff for infection risk. Among renal function indicators, serum creatinine ≥106 μmol/L remained independently associated with urinary tract infection (OR = 1.84, 95% CI: 1.09–3.11, *p* = 0.023). In contrast, the effect of serum urea nitrogen was attenuated in the multivariate model, suggesting that it may not be an independent risk factor.

**Table 6:** Variable assignment table.

Independent variable	Assignment
Hydronephrosis	No = 0; Yes = 1
Urinary tract obstruction	No = 0; Yes = 1
Urine pH	<7.0 = 0; ≥7.0 = 1
Stone diameter	<3 mm = 0; ≥3 mm = 1
Serum urea nitrogen	<7.5 mmol/L = 0; ≥7.5 mmol/L = 1
Serum creatinine	<106 μmol/L = 0; ≥106 μmol/L = 1

OR, odds ratio; CI, confidence interval.

**Table 7:** Multivariate logistic regression analysis of risk factors for urinary tract infection in patients with urolithiasis.

Factor	*β*	SE	*Wald*	*p*	OR	95% CI
Hydronephrosis	0.86	0.29	8.79	0.003	2.36	1.34–4.17
Urinary tract obstruction	0.71	0.27	6.86	0.009	2.03	1.20–3.43
Urine pH ≥ 7.0	0.94	0.28	11.28	0.001	2.56	1.48–4.45
Stone diameter ≥3 mm	0.68	0.26	6.73	0.010	1.97	1.17–3.33
Serum urea nitrogen ≥7.5 mmol/L	0.52	0.25	4.33	0.037	1.41	0.93–2.76
Serum creatinine ≥106 μmol/L	0.61	0.27	5.17	0.023	1.84	1.09–3.11

## 4 Discussion

Urinary calculi complicated by UTI represent a common condition in urological practice and are associated with potentially severe clinical consequences. Such infections not only aggravate patient symptoms but may also lead to infection spread, renal function impairment, and even sepsis (15, 16). Using retrospective data from a single center, this study evaluated factors associated with UTI in patients with urolithiasis and described the pathogen spectrum and antimicrobial resistance characteristics among infected patients. These findings may support infection prevention and rational antimicrobial use in clinical practice. Regarding pathogen composition, the results demonstrated that Gram-negative bacteria remained the predominant causative pathogens in patients with urolithiasis complicated by UTI, accounting for 68.54% of all isolates, with *Escherichia coli* and *Pseudomonas aeruginosa* being the most common species. This distribution pattern is largely consistent with previous studies on complicated UTIs (17, 18), suggesting that enteric opportunistic pathogens continue to play a dominant role in urinary tract infections.

The presence of urinary calculi provides an ideal physical substrate for bacterial adhesion and biofilm formation, particularly under conditions of urinary tract obstruction or urinary retention, which further facilitates the colonization and persistent proliferation of Gram-negative bacteria (19, 20). Meanwhile, Gram-positive bacteria accounted for 27.53% of isolates in this study, predominantly *Enterococcus faecalis* and *Enterococcus faecium*, indicating that opportunistic pathogens such as enterococci should not be overlooked in stone-associated infections. This may be related to repeated urinary tract manipulation, long-term indwelling catheterization, or prior antibiotic exposure (21). Antimicrobial resistance analysis further revealed the therapeutic challenges associated with stone-related UTIs. The results showed that E. coli exhibited relatively high resistance rates to ampicillin, cefazolin, and ceftazidime, while maintaining good susceptibility to imipenem, suggesting a declining effectiveness of traditional *β*-lactam antibiotics in empirical therapy. *Pseudomonas aeruginosa* demonstrated marked resistance to multiple commonly used antibiotics but remained highly sensitive to carbapenems. This phenomenon has been reported in several studies on complicated UTIs (22, 23) and may be attributed to intrinsic efflux pump mechanisms and β-lactamase production in this organism. Regarding Gram-positive bacteria, both *Enterococcus faecalis* and *Enterococcus faecium* showed high resistance rates to gentamicin, erythromycin, and penicillin G, while remaining susceptible to glycopeptide antibiotics such as vancomycin and teicoplanin. These findings suggest that empirical use of traditional aminoglycosides or macrolides should be avoided in suspected enterococcal infections. Collectively, these results highlight the importance of standardized pathogen identification and antimicrobial susceptibility testing in patients with urolithiasis complicated by infection, in order to reduce empirical treatment failure and prevent further dissemination of resistant strains. The additional annual trend analysis further supported the need for continuous antimicrobial resistance surveillance. During the study period, *Escherichia coli* maintained high resistance rates to ampicillin, cefazolin, and ceftazidime, while *Pseudomonas aeruginosa* showed persistently complete resistance to cefazolin. Although resistance rates to some agents fluctuated between years, no clear continuous increase in carbapenem resistance was observed, and no imipenem-resistant isolates of *Escherichia coli* or *Pseudomonas aeruginosa* were detected. However, given the limited number of isolates in each calendar year, especially in 2025, these temporal findings should be interpreted as descriptive trends rather than definitive evidence of long-term resistance evolution. The additional evaluation of MDR, ESBL-producing Enterobacterales, carbapenem-resistant organisms, and annual resistance trends further emphasizes the public health importance of continuous local antimicrobial resistance surveillance in patients with urolithiasis-associated UTI. When compared with international and regional antimicrobial resistance data, the present findings show both consistency and local specificity. Global surveillance reports have emphasized that Gram-negative pathogens, particularly *Escherichia coli* and *Klebsiella pneumoniae*, remain major contributors to UTI-related antimicrobial resistance worldwide (24). Asian surveillance data have also reported substantial resistance of *Escherichia coli* urinary isolates to fluoroquinolones and cephalosporins, supporting the need for local susceptibility-guided antimicrobial selection (25). In the present cohort, *Escherichia coli* showed high resistance to ampicillin, cefazolin, ceftazidime, and ciprofloxacin, which is generally consistent with these regional resistance concerns. However, no imipenem-resistant *Escherichia coli* or *Pseudomonas aeruginosa* isolates were detected, suggesting that carbapenem susceptibility was relatively preserved in this single-center cohort. Recent studies focusing on urolithiasis-associated UTI have similarly reported that *Escherichia coli* remains the most common pathogen and that hydronephrosis, obstruction, and alkaline urine are closely associated with infection risk (26). These comparisons indicate that the pathogen spectrum and risk factors observed in this study are broadly consistent with recent evidence, while the specific resistance profile should be interpreted in the context of local antimicrobial use and microbiological surveillance practices.

In terms of risk factor analysis, this study systematically evaluated factors associated with the occurrence of UTI in patients with urolithiasis through univariate and multivariate analyses. Multivariate logistic regression analysis identified hydronephrosis, urinary tract obstruction, elevated urine pH, stone diameter ≥3 mm, and elevated serum creatinine as independent risk factors for UTI. These factors collectively reflect the synergistic effects of urinary anatomical abnormalities, alterations in urinary physicochemical properties, and renal function impairment on infection development. Both hydronephrosis and urinary tract obstruction were associated with a markedly increased risk of infection in this study, with odds ratios exceeding 2, indicating that impaired urinary drainage represents a key pathological basis for stone-related infections. Urinary tract obstruction leads to urine retention, reduces the flushing effect of urine flow on bacteria, and creates favorable conditions for bacterial adhesion, biofilm formation, and sustained proliferation (27). In addition, prolonged high-pressure states may disrupt local mucosal barriers and weaken host defense mechanisms, thereby increasing susceptibility to infection (28). Previous studies (29) have also reported that obstructive calculi, if not promptly relieved, are more likely to progress to complicated infections or even sepsis. Elevated urine pH was also confirmed as an independent risk factor in this study. Alkaline urine not only favors the growth of certain urease-producing bacteria (such as *Proteus* spp.) but may also promote the formation of infection-related stones, resulting in a vicious cycle of “stone–infection–stone” (30). Although the detection rate of *Proteus* species was relatively low in this study, urine alkalization may still increase infection risk by altering the urinary microenvironment and enhancing the survival capacity of various opportunistic pathogens (31). The association between larger stone diameter and increased UTI risk also has important mechanistic implications. Larger calculi may cause more pronounced urinary tract irritation and greater disturbance of urine flow. More importantly, their irregular surfaces and larger surface area may provide a broader ecological niche for bacterial adhesion and biofilm formation (32). Once embedded within a biofilm matrix, bacteria may be protected from host immune clearance and may have reduced exposure to antimicrobial agents, thereby contributing to persistent infection and apparent antimicrobial resistance. This mechanism may partly explain why larger stones are more frequently associated with infection and are more difficult to eradicate (33). Therefore, in patients with a larger stone burden, infection control should not rely solely on antimicrobial therapy, but should also emphasize timely relief of obstruction and definitive stone management when clinically feasible. In contrast, stone location was not significantly different between patients with and without UTI in this cohort, suggesting that stone burden and urinary drainage impairment may be more relevant to infection risk than anatomical location alone. Furthermore, elevated serum creatinine remained statistically significant in the multivariate analysis, indicating that impaired renal function independently contributes to the development of stone-associated UTIs. Renal dysfunction may reflect prolonged urinary tract obstruction, recurrent infections, or heavy stone burden, and may also indirectly increase infection risk by affecting host immune status and the pharmacokinetics of antimicrobial agents (34, 35). In contrast, serum urea nitrogen did not emerge as an independent risk factor in the multivariate model, suggesting that it may primarily reflect short-term metabolic status rather than being a decisive determinant of infection occurrence.

Overall, this study provides a relatively comprehensive elucidation of the mechanisms underlying urolithiasis complicated by UTI from both pathogen-related and risk factor perspectives. The findings suggest that clinical management of patients with urinary calculi should extend beyond stone removal alone and emphasize comprehensive evaluation of urinary tract patency, urinary physicochemical parameters, and renal function status. For patients with hydronephrosis, urinary tract obstruction, alkaline urine, large stone burden, or renal dysfunction, heightened awareness of infection risk is warranted. Early interventions, including prompt relief of obstruction, optimization of the urinary environment, and rational use of antimicrobial agents, should be implemented to reduce the incidence of UTIs and improve patient prognosis. From a clinical translation perspective, the independent risk factors identified in this study may provide a preliminary framework for early UTI risk stratification in patients with urolithiasis. Patients presenting with multiple risk factors, such as hydronephrosis, urinary tract obstruction, alkaline urine, larger stone diameter, and elevated serum creatinine, should be considered at higher risk for UTI and may benefit from closer monitoring, earlier urine culture testing, timely relief of obstruction, and individualized antimicrobial decision-making. Although these factors could serve as candidate predictors for a future risk prediction model or nomogram, the present study did not construct a formal nomogram because external validation and model performance assessment were not available. Future multicenter prospective studies are needed to develop and validate a clinically applicable prediction tool.

## 5 Study limitations and future perspectives

This study evaluated UTI-related clinical factors, pathogen distribution, and antimicrobial resistance characteristics in patients with urolithiasis. Although clinically relevant findings were obtained, several limitations should be acknowledged. (1) This study adopted a retrospective design, and the clinical data were derived from existing medical records. Although efforts were made to minimize information loss and selection bias through strict inclusion criteria and standardized data extraction procedures, some potential confounding factors could not be fully controlled. In addition, although patients who had received antimicrobial therapy within 2 weeks before urine testing were excluded to reduce the influence of recent antibiotics on culture and susceptibility results, this criterion may have introduced selection bias. Patients with more severe, recurrent, or previously treated infections may have been underrepresented. Therefore, the pathogen distribution and antimicrobial resistance profiles observed in this study may mainly reflect patients without recent antimicrobial exposure and may not fully represent all clinical cases of urolithiasis-associated UTI. In addition, retrospective studies are inherently limited in establishing causal relationships; therefore, the identified risk factors in this study primarily reflect statistical associations, and their causal relationships require further confirmation through prospective studies. Moreover, because this was a retrospective study based on all eligible cases within a fixed study period, a formal prospective sample size calculation with prespecified *α* and *β* values was not performed before patient enrollment. Future prospective studies should include *a priori* sample size estimation based on predefined primary outcomes. (2) As a single-center study, the generalizability of the present findings may be limited. The patient population was derived from one hospital and may have specific regional, demographic, and clinical characteristics. In addition, pathogen distribution and antimicrobial resistance profiles are strongly influenced by local antimicrobial prescribing practices, healthcare-associated infection control measures, and microbiological surveillance patterns. Therefore, caution is needed when applying the present findings to other regions or healthcare settings. Future multicenter studies involving different levels of hospitals and diverse geographic regions are needed to externally validate these risk factors and resistance patterns, thereby improving the robustness and applicability of the conclusions. (3) Regarding pathogen analysis, this study was primarily based on conventional urine culture and antimicrobial susceptibility testing. Further molecular analyses, such as resistance gene detection, molecular typing, or biofilm-forming capacity assessment, were not performed. Given the close association between stone-related UTI and biofilm formation, future studies should incorporate biofilm-related markers and molecular microbiological techniques to better clarify the mechanisms linking urolithiasis, persistent infection, and antimicrobial resistance. In addition, antimicrobial susceptibility interpretation was based on the original CLSI 2018 criteria recorded in the laboratory information system, and the results were not retrospectively reinterpreted using newer CLSI breakpoints. Therefore, direct comparison with studies using more recent CLSI standards should be made cautiously. (4) In the analysis of stone characteristics, this study mainly focused on stone number, maximum diameter, and anatomical location. Stone composition was not systematically analyzed because standardized stone component testing was not routinely performed for all included patients. Therefore, patients could not be reliably classified into infection-related stone or metabolic stone groups, and stone composition could not be incorporated into the regression analysis. In addition, stone diameter was analyzed using a categorical threshold, and sensitivity analyses using alternative cutoffs or continuous stone size variables were not performed. These factors may limit the interpretation of the relationship between stone characteristics and UTI risk. Future prospective studies with complete stone composition data and more detailed stone size measurements are needed to further clarify how different stone types and stone burden are associated with infection risk. (5) Clinical outcome data were not systematically analyzed in the present study. Outcomes such as sepsis, intensive care unit (ICU) admission, length of hospitalization, in-hospital mortality, and stone recurrence were not included as predefined endpoints because the primary focus of this study was to evaluate UTI risk factors, pathogen distribution, and antimicrobial resistance characteristics. In addition, some outcome variables, particularly stone recurrence, require standardized follow-up data, which were not uniformly available in this retrospective dataset. Therefore, the clinical prognostic impact of UTI and antimicrobial resistance in patients with urolithiasis could not be fully assessed. Future prospective studies should incorporate short-term and long-term clinical outcomes to further clarify how UTI, pathogen type, and antimicrobial resistance affect patient prognosis and stone recurrence.

Despite these limitations, this study integrated clinical, microbiological, and antimicrobial resistance data from patients with urolithiasis, providing useful evidence for local infection surveillance and risk assessment. Future prospective multicenter studies should further validate these findings and develop risk prediction models based on key clinical predictors, such as hydronephrosis, urinary tract obstruction, urine pH, stone burden, and renal function indicators.

## 6 Conclusion

This study evaluated pathogen distribution, antimicrobial resistance patterns, and UTI-related clinical factors in patients with urolithiasis using single-center retrospective data. The results demonstrated that Gram-negative bacteria were the predominant pathogens in urinary calculi complicated by urinary tract infection, with *Escherichia coli* and *Pseudomonas aeruginosa* occupying the leading positions. Significant differences in resistance profiles were observed among different pathogens, indicating inherent limitations of empirical antimicrobial therapy and underscoring the importance of individualized antibiotic selection guided by pathogen identification and antimicrobial susceptibility testing. Risk factor analysis revealed that hydronephrosis, urinary tract obstruction, elevated urine pH, larger stone diameter, and increased serum creatinine levels were independent risk factors for urinary tract infection in patients with urolithiasis.

These factors elucidate the potential mechanisms underlying stone-related infection from multiple dimensions, including urinary tract anatomy, urinary microenvironment, and renal function status, and emphasize the importance of comprehensive infection risk assessment during stone management.

In summary, clinical management of patients with urolithiasis should not only focus on active treatment of the stones themselves but also strengthen infection risk identification and intervention for high-risk patients. Timely relief of urinary tract obstruction, optimization of the urinary environment, and rational selection of antimicrobial agents based on microbiological evidence are essential to reduce the incidence of urinary tract infection and improve patient prognosis. The findings of this study may provide useful reference for clinical prevention strategies and antimicrobial treatment decision-making in patients with urolithiasis complicated by urinary tract infection.

## Data Availability

The original contributions presented in the study are included in the article/supplementary material, further inquiries can be directed to the corresponding author/s.
